# Diagnostic and prognostic value of MR-pro ADM, procalcitonin, and copeptin in sepsis

**DOI:** 10.1515/med-2023-0865

**Published:** 2023-12-31

**Authors:** Basar Cander, Emin Fatih Visneci, Osman Karaoglan, Fatma Cakmak, Alpay Tuncar, Bahadir Taslidere

**Affiliations:** Department of Emergency Medicine, Bezmialem Vakif University, Istanbul, Turkey; Department of Emergency Medicine, Konya Numune Hospital, Konya, Turkey; Department of Emergency Medicine, Konya City Hospital, Konya, Türkiye; Department of Emergency Medicine, Erzurum City Hospital, Erzurum, Türkiye

**Keywords:** sepsis, MR-proADM, copeptin, procalcitonin

## Abstract

Sepsis is defined as life-threatening organ dysfunction caused by a dysregulated host response to infection. There is a need for biomarkers that can be used for the diagnosis of sepsis and the early identification of patients at high risk of death. In this study, we aimed to investigate the relationship between Mid-regional pro-adrenomedullin (MR-proADM), procalcitonin (PCT), and copeptin in sepsis. A total of 28 sepsis, 32 septic shock, and 30 control patients were included in our prospective study. Patients’ MR-proADM, PCT, and copeptin levels were recorded. Sequential organ failure assessment scores, length of hospital stay, and 30-day mortality were also recorded. These values were compared between the sepsis, septic shock, and control groups. The mean age of all participants was 64.04 ± 15.83 years. In the study, 37 (61.6%) patients were female and 23 (39.3%) were male. There was no statistically significant difference in gender/age between all patient groups and the control group (for all, *p* > 0.05). We found a significant difference between the survivors and nonsurvivors in terms of MR-proADM, PCT, and copeptin levels. There was a significant difference between the sepsis and septic shock groups in terms of MR-proADM and PCT. A significant correlation was found between the length of hospital stay and MR-proADM and copeptin. MR-proADM, PCT, and copeptin may be useful in the prognosis of sepsis and to predict the length of stay in hospital and mortality.

## 1 Introduction

Sepsis is defined as life-threatening organ dysfunction caused by a dysregulated host response to an infection [1,2,3]. Septic shock is the last and most severe stage of sepsis. The mortality rate is about 50–60% [4,5]. None of the clinical signs and symptoms traditionally used in the diagnosis of sepsis and laboratory findings are unique to sepsis. Therefore, there is a need for biomarkers that can be used for the diagnosis of sepsis and the early identification of patients [6,7].

Mid-regional pro-adrenomedullin (MR-proADM), the middle part of pro-adrenomedullin, consists of 45–92 amino acids and directly reflects plasma adrenomedullin (ADM) levels [8]. Circulating levels of bioactive adrenomedullin (BioADM) can be measured using new technology and indicate vascular dysfunction occurring in sepsis. In this way, physiopathological deterioration in sepsis can be detected and progression to septic shock can be predicted [9]. ADM levels increase in sepsis, and MR-proADM has recently been shown to be a helpful prognostic tool in risk stratification in sepsis [10].

Procalcitonin (PCT) may aid in the diagnosis and management of patients. PCT is a useful biomarker in severe bacterial infection. The sensitivity of PCT in sepsis ranges from 42 to 97%, and the specificity ranges from 48 to 100% [7].

Arginine vasopressin (AVP) is one of the most important hypothalamic stress hormones [11].

Copeptin is the C-terminal part of the prohormone of AVP or antidiuretic hormone [12]. Measurement of AVP is not commonly carried out in clinical practice. In contrast, copeptin can be immunologically tested with ease [13]. Copeptin increases rapidly in the plasma in conditions such as cardiovascular diseases, ischemic stroke, sepsis, and shock, and this increase shows that AVP release is increased and has diagnostic and prognostic value [14]. There is no gold standard method as a biochemical marker in the diagnosis and prognosis of sepsis. However, it is important to make a rapid diagnosis and start treatment early. In this study, we aimed to investigate the diagnostic and prognostic values of MR-proADM, PCT, and copeptin in sepsis.

## 2 Materials and methods

This is a single-center, prospective controlled study of sepsis patients visiting the Emergency Department (a tertiary care teaching hospital). A consecutive sampling method was used. This research was carried out between 01/12/2017 and 01/06/2018. Patients were selected according to the International Sepsis Conference criteria [1]. The criteria for inclusion were the fulfillment of two of four criteria for the systemic inflammatory response syndrome (fever or hypothermia, tachycardia, tachypnea, and leukocytosis or leucopenia) and systolic blood pressure no higher than 90 mm Hg (after a crystalloid-fluid challenge). Necessary consent was obtained from the patients and control group. Those who did not want to contribute to the conduct of the study were excluded from the study. Exclusion criteria were those under 18 years of age, pregnant women, stroke, myocardial infarction, pulmonary edema, seizure, trauma, need for emergency surgery, cancer, non-bacterial pneumonia, etc. Patients who were similar in age and gender and had no signs of infection were selected as the control group. A total of 28 sepsis, 32 septic shock, and 30 control patients were included in our study over a six-month period. The control group was compared with both patient groups (sepsis and septic shock) separately. The patient group was identified, and cases were randomly selected according to specific inclusion criteria.

Ethics committee approval was obtained. The study was carried out in accordance with the Declaration of Helsinki.

### 2.1 Data collection

Patients’ demographic data were recorded when they were admitted to the emergency. Vital parameters (blood pressure, fever, pulse) and laboratory results (white blood cell, hemoglobin, hematocrit, platelet, urine output, urea, creatinine, sodium, potassium, total bilirubin, blood gas) were recorded on the forms. Sequential organ failure assessment (SOFA) scores of the patients were calculated. Length of hospital stay and 28-day mortality were recorded for the sepsis and septic shock groups. The mortality status of the discharged patients was learned by following the new admissions to the hospital and by telephone on the 30th day. Following data collection, two emergency attending physicians independently reviewed the medical records

### 2.2 Biochemical analysis

Blood samples were obtained from all recruited patients within 24 h of presentation to the hospital by an indwelling arterial or venous catheter (if available) or by venipuncture. Patients with septic shock also had blood drawn every 24 h up to 120 h after admission. After the patients were diagnosed with sepsis or septic shock (a blood sample was taken before giving antibiotics), 3 cc peripheral venous blood was taken into tubes (with ethylenediaminetetraacetic acid [EDTA]). Blood samples were centrifuged (3,000 rpm for 10 min) within the first hour after collection and stored at −80°C. The same procedure was applied in the control group. MR-proADM was measured in EDTA-K2 plasma samples using the BRAHMS Kryptor Compact Plus method (Hennigsdorf, Germany). Copeptin was evaluated by Time-Resolved Amplified Cryptate Emission on the ultra-sensitive Kryptor compact Plus (Henningsdorf, Germany). PCT levels in serum were measured using reagents from Thermo Fisher Scientific.

### 2.3 Statistical analysis

MR-proADM, PCT, and Copeptin values of the patient and control groups were compared. MR-proADM, PCT, and Copeptin values were compared in both patient groups (*p*-value 1, 2, 3). Data obtained in the study were statistically analyzed using the Statistical Package for Social Sciences software. Data were expressed as frequency (*n*), percentage (%), and mean ± standard deviation. The comparison of the distribution of categorical data was carried out with the “Chi-square (*χ* ^2^) test”. The normality of the continuous variables was evaluated using the Kolmogorov–Smirnov test. The comparison of normally distributed parameters between more than two groups was made using a one-way analysis of variance. The comparison of non-normally distributed parameters between the two groups was performed with the Mann–Whitney *U* test, and the comparison between more than two groups was performed with the Kruskal–Wally’s test. Correlations between the parameters were examined using Spearman’s analysis. *p* < 0.05 values were considered statistically significant. A receptor operating characteristic (ROC) curve analysis was applied for biomarkers.

## 3 Results

The mean age of all participants was 64.04 ± 15.83 years. The mean age of all participants was 64.04 ± 15.83 years. The mean age of the sepsis group was 63.5 ± 16.11 years, the septic shock group was 64.1 ± 14.3 years, and the control group was 64 ± 15.34 years. There were 37 females (61.6%) and 23 males (39.3%) in the patient group, and 17 females (56.7%) and 13 males (43.3%) in the control group. There was no statistically significant difference in gender/age between all patient groups and the control group (for all, *p* > 0.05) (Table 1).

**Table 1:** Demographic, laboratory, and comorbidity

		Sepsis (*n* = 28)	Septic shock (*n* = 32)	Control (*n* = 30)
Age	Year	63.58 ± 13.9	64.06 ± 10.32	64.04 ± 15.83
PCT	ng/mL	1.01 ± 4.11	15 ± 52.04	0.53 ± 3.73
MR-proADM	nmol/L	2.19 ± 2.60	3.70 ± 8.90	1.07 ± 2.50
Copeptin	pmol/L	67.5 ± 42.6	126 ± 245	51 ± 188
Gender	Female, *n* (%)	37 (61.6)	17 (56.7)	
	Male, *n* (%)	23 (39.3)		13 (43.3)
DM	*n* (%)	15 (25)		3 (10)
HT	*n* (%)	12 (20)		5 (16.6)
COPD	*n* (%)	6 (10)		4 (13)
Malignancy	*n* (%)	20 (33)		7 (23)
CAD	*n* (%)	5 (8.3)		3 (10)
Other	*n* (%)	6 (10)		4 (13)

DM = diabetes mellitus, HT = hypertension, COPD = chronic obstructive pulmonary disease, CAD = coronary artery disease.

When the patients with sepsis were examined in terms of the infection focus, lung-based infection was observed, 26 (43.39%), urinary tract infection, 16 (26.6%), soft tissue infection, 10 (16.6%), intra-abdominal infection, 4 (6.6%), central nervous system infection, 3 (5%), and cardiac infection, 1 (1.6%).

When the mean MR-proADM values were evaluated; there were significant differences between the sepsis and control groups, the septic shock and control groups, and the sepsis and septic shock groups (*p* = 0.02, *p* < 0.01, *p* = 0.04, respectively) (Table 2).

**Table 2:** Mean MR-proADM and PCT values of the groups

	Sepsis (*n* = 28)	Septic shock (*n* = 32)	Control (*n* = 30)	*p* ^1^	*p* ^2^	*p* ^3^
MR-proADM	4.8 ± 7.7	7.2 ± 7.1	2.1 ± 2.5	0.04	0.02	<0.01
PCT	10.6 ± 25.6	15.5 ± 34.6	1.6 ± 3.7	>0.05	0.01	<0.01
Copeptin	67.5 ± 42.6	126 ± 245	51 ± 188	>0.05	>0.05	>0.05

*p* ^1^ values = between sepsis and septic shock, *p* ^2^ = between sepsis and control group, and *p* ^3^ = between septic shock and control group.

There was no significant difference between the sepsis and septic shock groups in terms of mean PCT value (*p* > 0.05). However, a statistically significant difference was found between the sepsis and control groups and between the septic shock and control groups (*p* = 0.05, *p* < 0.01, respectively) (Table 2).

When the mean copeptin values were evaluated; there was no significant difference between the sepsis, septic shock, and control groups in terms of the mean copeptin values (for all, *p* > 0.05) (Table 2).

When the correlation between mortality and biomarkers was evaluated, 32 of our patients were alive and 28 died. The mean MR-proADM value of the patients who survived was 3 nmol/L and the mean MR-proADM value of the patients who died was 5.12 nmol/L. There was a significant difference between the MR-proADM values of the patients who survived and nonsurvivors (*p* < 0.05). The mean PCT value of the patients who survived was 1.82 ng/mL and the mean PCT value of the patients who died was 13.85 ng/mL. There was a significant difference between the PCT values of the patients who survived and nonsurvivors (*p* < 0.05). The mean copeptin value of the patients who survived was 73 pmol/L and the mean copeptin value of the patients who died was 142 pmol/L. There was a significant difference between the copeptin values of the patients who survived and nonsurvivors (*p* < 0.05) (Table 3). Considering the correlation of hospital stays with biomarkers; a significant correlation was found between the MR-proADM and copeptin and the length of stay in hospital (both, *p* < 0.05) (Table 4). Significant correlations were found between the mean MR-proADM, PCT, and calcitonin levels and SOFA scores.

**Table 3:** The relationship between mortality and biomarkers

		*M* value	*p*-value
PCT	A	1.82 ± 24.21 ng/mL	<0.05
	B	13.85 ± 51.43 ng/mL	
MR-proADM	A	3 nmol/L	<0.05
	B	5.12 nmol/L	
Copeptin	A	73 pmol/L	<0.05
	B	142 pmol/L	

A = survivors, B = non-survivors, and M = median.

**Table 4:** Correlation of biomarkers with the length of stay in hospital

Biomarker	***r***	*p*-value
MR-proADM	0.403	<0.01
PCT	0.202	>0.05
Copeptin	0.398	<0.01

*r* = correlation coefficient (among all patients).

**Figure 1:**
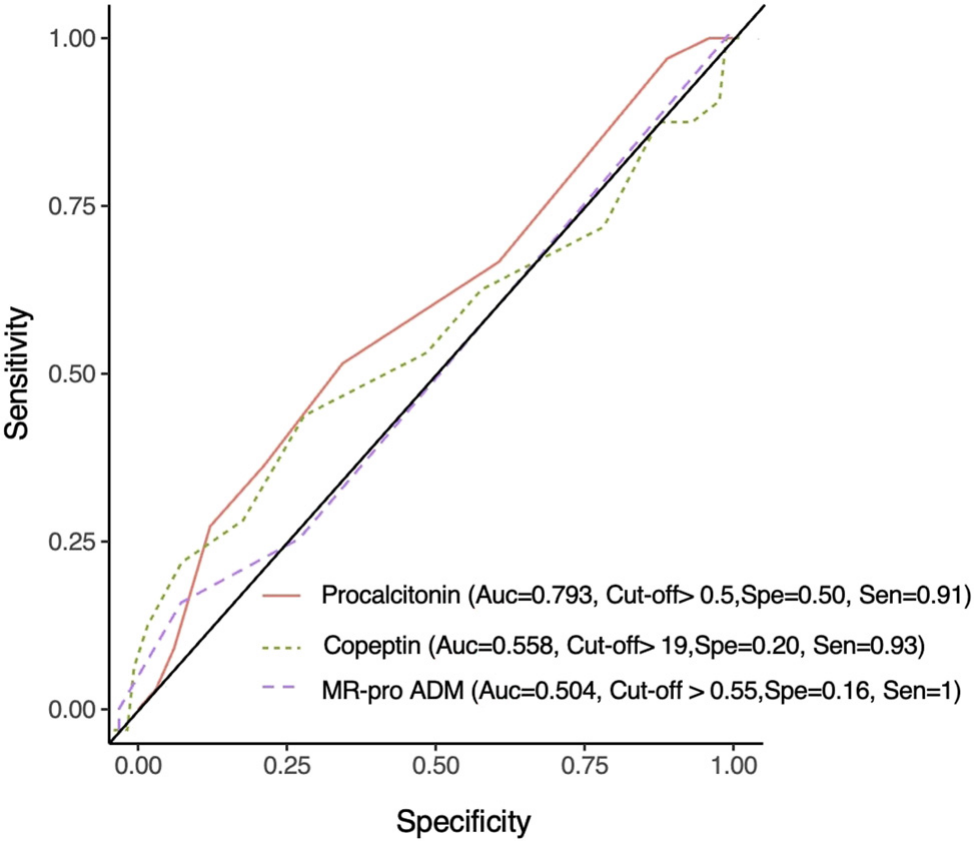
ROC curves.

The sensitivity, specificity, positive predictive value (PPV), AUC, and negative predictive value (NPV) of the biomarkers examined in this study are given in Table 5. The data obtained are described in Figure 1 with the ROC curve.

**Table 5:** Sensitivity, specificity PPV, and NPV of biomarkers in all patients

Biomarker	Sen. (%)	Spe. (%)	PPV (%)	NPV (%)	AUC	Cut-off
MR-proADM	100	16.60	70	100	504	0.55 nmol/L
PCT	91.30	50	78.50	75	793	0.5 ng/mL
Copeptin	93	20	70	60	558	19 pmol/L

NPV = negative predictive value, PPV = positive predictive value, Sen = sensitivity, Spe = specificity.

## 4 Discussion

According to the latest guidelines, treatment for sepsis should be individualized, and biomarkers should be used to tailor therapy to each patient’s needs [15,16]. Sepsis is a disease with high mortality and morbidity, and treatment should be initiated quickly. There is a need for an inexpensive, easily obtainable biomarker with high sensitivity and specificity in sepsis. Peptides are not just markers, peptides can also serve as mediators. Therefore, usage areas may differ [17,18]. In a study by Lai et al., the most common bacterial infection was pneumonia (40.2%) followed by bacteremia and urinary tract infections (23.5%), intra-abdominal infections (16.2%), and skin and soft tissue infections (14.2%) [19]. Similarly, in our study, the most common infection was pneumonia followed by urinary tract infection, soft tissue infection, and intra-abdominal infection. We think that this may be due to the increased incidence of ventilator-associated pneumonia due to the more frequent use of mechanical ventilators in patients followed up in the intensive care unit in recent years.

BioADM is a biomarker, which has been used for vascular and endothelial function and utility in sepsis [20]. We found that significant differences were found between the sepsis and septic shock groups and the control group in terms of MR-pro-ADM, and we were able to predict the prognosis, mortality, and length of stay in hospital using MR-pro-ADM.

Gille et al. evaluated copeptin levels in sepsis after burn injury and found no significant difference between patients with and without sepsis [21]. One study reported that Copeptin levels were higher than survivors in non-surfing [7]. In our study, in line with the literature, no statistically significant difference was found between the patients with sepsis or septic shock, and the control group, in terms of the levels of copeptin, which is released in equal amounts with AVP and is a more stable molecule in the blood. However, there was a significant difference between survivors and nonsurvivors.

In a recent review by Di Somma and Crisanti with 773 patients investigating biomarkers in the management of sepsis, the median PCT value was found to be significantly higher in patients with culture-positive sepsis than in those with culture-negative sepsis [9]. In our study, there were significant differences between sepsis, septic shock, and control groups regarding PCT values.

In a study by Novotny et al., the sepsis-related mortality rate was 36%. PCT levels of nonsurvivors were found to be significantly higher than survivors. The authors concluded that PCT levels are an independent predictor of mortality in patients with sepsis [22]. In our study, we found a 30-day mortality rate of 46.6%. MR-proADM, PCT, and copeptin levels were compared between the patients who survived and nonsurvivors. Consistently with the literature, we found a significant difference between the survivors and nonsurvivors in terms of MR-proADM values. In addition, statistically significant differences were found between the survivors and nonsurvivors in terms of PCT and copeptin values. In the present study, we found a significant difference between the sepsis and septic shock groups in PCT. In light of these findings, we think that critical patients with high MR-proADM, PCT, and copeptin values have a higher mortality rate and these patients should be followed more closely. We think that these markers can be used to determine mortality.

In a study by Schneider et al. including 220 patients with sepsis, the median length of stay in the intensive care unit was found as 13 (4–95) days in the survivors. The median PCT values measured on the first postoperative day were found to be significantly higher in patients who died than in those who survived. In the same study, it was emphasized that there was a significant correlation between high PCT concentrations and prolonged hospital stay [23].

In our study, when the correlation between the length of hospital stay and PCT, copeptin, and MR-proADM was examined, no significant correlation was found between PCT and hospitalization time, unlike the literature. A significant correlation was found between the length of hospital stay and MR-pro ADM and copeptin. Both acute physiology and SOFA scores are widely used in critically ill patients [22]. In our study, SOFA scores were significantly correlated with the mean MR-pro ADM, PCT, and copeptin values, consistently with the literature [25,26].

In a study by Schneider et al., the median length of stay in the intensive care unit was 13 (4–95) days for the survivors [23]. In our study, when the correlation between the length of hospital stays and PCT, copeptin, and MR-proADM were examined, no significant correlation was found between PCT and hospitalization time, unlike the literature (*p* > 0.05). A significant correlation was found between the length of hospital stay and MR-pro ADM and copeptin (*p* < 0.05). SOFA scores are widely used to predict the outcome of critically ill surgical patients including those with sepsis [24]. In our study, SOFA scores were significantly correlated with the mean MR-pro ADM, PCT, and copeptin values, consistently with the literature [25,26].

In a study by Tsangaris et al., PCT concentration was 1.00 mg/L in sepsis patients and 0.18 mg/L in the controls. When the upper cut-off value for PCT was determined as 1.00 ng/mL, the sensitivity was 70%, specificity 91%, PPV 90%, and NPV 72% for patients with sepsis [27]. In our study, when the cut-off value for PCT was taken as 0.5 ng/mL, the sensitivity of PCT for the diagnosis of sepsis was 91.3%, specificity 50%, PPV 78.5%, and NPV 75%. When the cut-off value for copeptin was taken as 19 pmol/L, the sensitivity of copeptin for the diagnosis of sepsis was 93%, specificity 20%, PPV 70%, and NPV 60%. When the cut-off value for MR-proADM was taken as 0.55 nmol/L, the sensitivity of MR-proADM for the diagnosis of sepsis was 100%, specificity 16.6%, PPV 70%, and NPV 100%. In our study, although the sensitivity of copeptin and MR-proADM was high, the specificity was low.

## 5 Study limitations

We think that copeptin and MR-proADM values of the patients in the control group may have been measured to be high since it is still not known precisely in which comorbid diseases copeptin and MR-proADM values increase.

## 6 Conclusion

A significant difference was found between copeptin, PCT, and MR-proADM values in surviving and non-surviving sepsis patients. A significant correlation was found between MR-proADM and copeptin and length of hospital stay. We think that MR-proADM, PCT, and copeptin may be used in predicting the prognosis of patients with sepsis, mortality, and length of stay in the hospital. Because of the complexity of the sepsis response, a single biomarker is unlikely to be sufficient. With its ability to respond quickly to clinical conditions, copeptin, PCT, and MR-proADM can be used for treatment follow-up and early diagnosis.
